# Laparoscopic Management of an Abdominal Pregnancy

**DOI:** 10.1155/2014/562731

**Published:** 2014-11-11

**Authors:** Aarthi Srinivasan, Suzanne Millican

**Affiliations:** Department of Obstetrics and Gynecology, St. John Hospital & Medical Center, 22151 Moross Road, Suite 320, Detroit, MI 48236, USA

## Abstract

*Background*. Ectopic pregnancy is one of the leading causes of significant maternal morbidity and mortality. Abdominal surgeries increase the risk of postoperative adhesions. We here present a case of omental ectopic pregnancy in a patient with a prior history of cesarean section. *Case*. A 20-year-old female presented with a two-day history of crampy lower abdominal pain. Patient was hemodynamically stable with a beta HCG of 1057 mI/mL. Transvaginal ultrasound did not show an intrauterine pregnancy but revealed an ill-defined mass in the midline pelvis extending to the right of the midline. Diagnostic laparoscopy revealed large clots in the pelvis with normal uterus and adnexa. Intra-abdominal survey revealed an omental adhesion close to the right adnexa with a hematoma. Partial omentectomy was completed and the portion of the omentum with the hematoma was sent to pathology for confirmation. Final pathology confirmed the presence of chorionic villi consistent with products of conception. *Conclusion*. Omental ectopic pregnancy is a rare diagnosis and often missed. We recommend careful intra-abdominal survey for an ectopic pregnancy in the presence of hemoperitoneum with normal uterus and adnexa. This can be safely achieved using laparoscopy in early gestational ages when the patient is hemodynamically stable.

## 1. Introduction

Ectopic pregnancy occurs when the blastocyst implants anywhere other than in the endometrial lining [1]. Ectopic pregnancy is one of the leading causes of significant maternal morbidity and mortality [1]. The incidence of ectopic pregnancy is 1-2% of pregnancies in the USA [2, 3]. With 90% of the ectopic pregnancies implanting in the fallopian tubes, abdominal pregnancies account for only 1.4% of the ectopic pregnancies [1]. They present a diagnostic challenge due to difficulty in identifying the implantation site. We here describe a case of an early abdominal pregnancy managed laparoscopically.

## 2. Case

**Figure 1:**
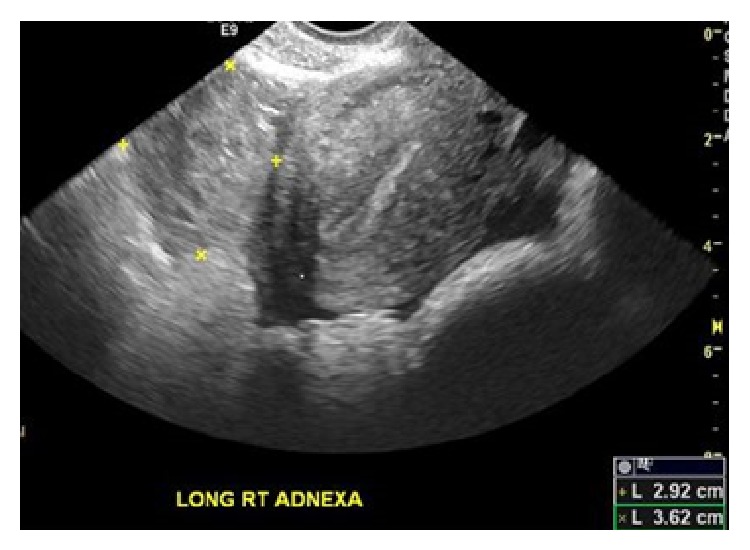
Mass-like area in the midline pelvis extending to the right of the midline measuring 2.9 × 3.8 × 3.6 cms.

**Figure 2:**
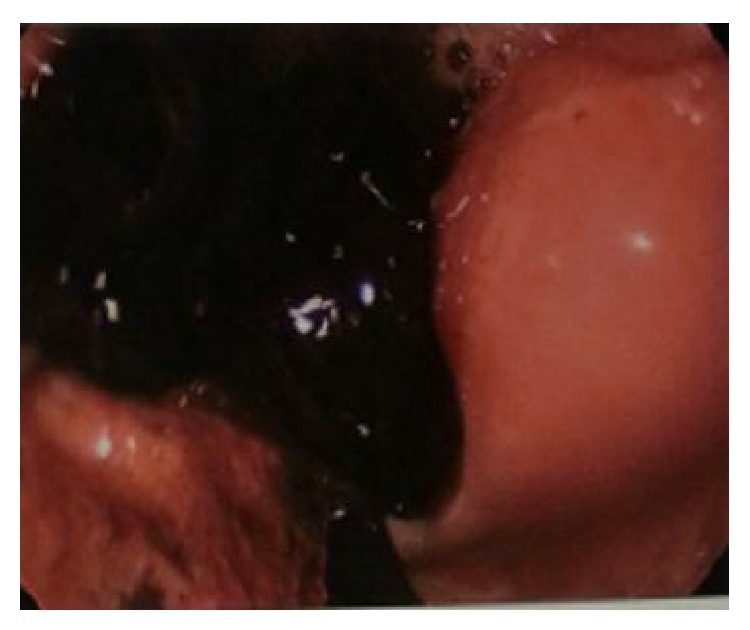
Hemoperitoneum noted during laparoscopy.

**Figure 3:**
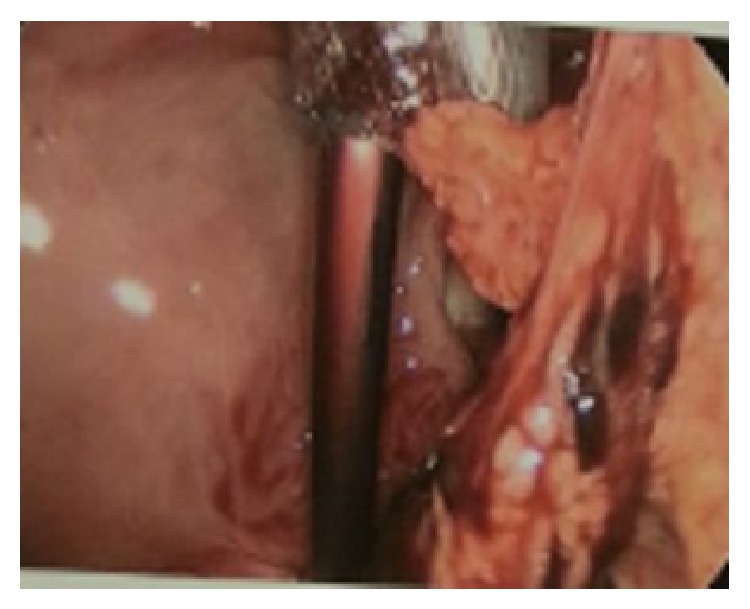
Omental adhesion close to the right adnexa with hematoma.

The patient is a 20-year-old African American female gravida 2, para 1 at 8-week-and-5-day gestational age based on her last menstrual period, who presented to the emergency department with a two-day history of crampy lower abdominal pain. She had a history of a full term cesarean section with her previous pregnancy. The patient rated her pain as 8 on a scale of 10. Physical examination revealed suprapubic and right lower quadrant tenderness, but there were no signs of acute peritonitis. She was hemodynamically stable with a blood pressure of 119/78 mmHg, hemoglobin of 11.4 mg/dL, and beta HCG of 1057 mIU/mL. Transvaginal ultrasound revealed no evidence of an intrauterine pregnancy but did show a moderate to large amount of complex collection of fluid in the anterior and posterior cul-de-sacs. Also noted was an ill-defined mass-like area in the midline pelvis and extending slightly to the right of midline measuring 2.9 × 3.6 × 3.8 centimeters (Figure 1). Bilateral ovaries were within normal limits with normal Doppler flow. The above findings were concerning for a ruptured ectopic pregnancy. In light of the patient's symptoms and clinical data, the patient was taken to the operating room for a diagnostic laparoscopy. On inspection, large clots of blood were noted in the pelvis (Figure 2). The uterus, bilateral tubes, and ovaries appeared normal. An intact corpus luteum was noted on the left ovary. However, on further exploring the abdomen, we noted an omental adhesion near the right fallopian tube (Figure 3). On inspection, the omentum was found to have a hematoma within it. There was no evidence of a tubal or ovarian pregnancy based on the normal appearing anatomy of the pelvis. At this time, our presumptive diagnosis was an omental ectopic pregnancy since there was no other explanation for the laparoscopic findings.

General surgery was consulted and a partial omentectomy was completed. The portion of the omentum with the hematoma was sent to pathology for confirmation of a pregnancy. The patient did well postoperatively and was discharged home on postoperative day 1. Beta HCG the following morning before discharge had dropped to 471 mIU/mL.

Follow-up beta HCG values on postoperative day 5 and day 12 were 45 mIU/mL and 4 mIU/mL, respectively. The patient did not receive methotrexate in the postoperative period. Final pathology confirmed the presence of chorionic villi consistent with products of conception with portions of adipose tissue and blood clot within the omentum.

## 3. Discussion

Primary abdominal pregnancy is an extremely rare form of ectopic pregnancy. The incidence is reported to be 1 in 10,000 live births [1, 2]. In 1942, Studdiford defined the criteria for abdominal pregnancy: normal bilateral fallopian tubes and ovaries, absence of uteroperitoneal fistula, and presence of a pregnancy related to the peritoneal surface exclusively [4]. Our patient here met the above diagnostic criteria as described by Studdiford. While pelvic inflammatory disease, assisted reproductive technologies, endometriosis, and tubal damage are some of the risk factors identified, our patient did not have any of these risk factors [5, 6]. Although most abdominal pregnancies are proposed to be secondary, an intact corpus luteum was noted here [7].

Weeks et al. in 1997 described a case of primary omental pregnancy diagnosed with laparoscopy at 6-week gestational age where a hemorrhagic mass was noted to be adherent to the omentum close to the left ovary [8]. Gerli et al. in 2003 reported a case of an abdominal pregnancy managed with laparoscopy where the gestational sac was noted in the pouch of Douglas [9]. A few other case reports have been found in the literature where a hemorrhagic mass with blood clots appears to be a consistent finding like in our case [10–12]. Exploratory laparotomy is recommended in most cases of abdominal pregnancies due to the risk of significant hemorrhage from the implantation site [13]. The use of laparoscopy has been described in the literature for the diagnosis and management of abdominal pregnancy [8–12]. Our patient was hemodynamically stable and hence we were able to perform laparoscopy safely without any complications

Intraperitoneal adhesions are one of the long term risks of abdominal surgeries. With one cesarean section, the prevalence of adhesions is 12 to 46% [14]. When the implantation occurs in the adhesions, the diagnosis can be easily missed due to difficulty in locating the pregnancy especially when it is a very early pregnancy. For patients who present with a history of prior abdominal surgeries, when the uterus or the adnexa do not reveal the implantation site, we stress the importance of surveying intraperitoneal adhesions for an ectopic pregnancy.

## 4. Conclusion

Abdominal pregnancy is frequently missed or the diagnosis is delayed. A high index of clinical suspicion is necessary for diagnosis. Only 40% of abdominal pregnancies are diagnosed before surgery [15]. This is where early prenatal ultrasound is helpful in identifying those cases of ectopic pregnancies. We were able to manage this patient successfully using laparoscopy and recommend the use of the same in patients with early abdominal pregnancies that are hemodynamically stable. In conclusion abdominal pregnancy is a rare event and should be considered as a possible cause for hemoperitoneum when no other source can be identified in the uterus or the adnexa.
